# Coffee consumption and bladder cancer: a meta-analysis of observational studies

**DOI:** 10.1038/srep09051

**Published:** 2015-03-12

**Authors:** Weixiang Wu, Yeqing Tong, Qiang Zhao, Guangxia Yu, Xiaoyun Wei, Qing Lu

**Affiliations:** 1 Key Laboratory of Environment and Health, Ministry of Education & Ministry of Environmental Protection, and State Key Laboratory of Environmental Health (Incubating), School of Public Health, Tongji Medical College, Huazhong University of Science and Technology, #13 Hangkong Road, Wuhan, Hubei. 430030, China; 2 Hubei provincial center for disease control and prevention

## Abstract

Controversial results of the association between coffee consumption and bladder cancer (BC) risk were reported among epidemiological studies. Therefore, we conducted this meta-analysis to clarify the association. Relevant studies were identified according to the inclusion criteria. Totally, 34 case-control studies and 6 cohort studies were included in our meta-analysis. The overall odds ratio (OR) with 95% confidence interval (CI) between coffee consumption and BC risk was 1.33 (95% CI 1.19 to 1.48). The summary ORs of BC for an increase of 1 cup of coffee per day were 1.05 (95% CI 1.03 to 1.06) for case-control studies and 1.03 (95% CI 0.99 to 1.06) for cohort studies. The overall ORs for male coffee drinkers, female coffee drinkers and coffee drinkers of both gender were 1.31 (95% CI: 1.08 to 1.59), 1.30 (95% CI: 0.87 to 1.96) and 1.35 (95% CI: 1.20 to 1.51). Compared with smokers (OR = 1.24, 95% CI: 0.91 to 1.70), non-smokers had a higher risk (OR = 1.72, 95% CI: 1.25 to 2.35) for BC. Results of this meta-analysis suggested that there was an increased risk between coffee consumption and BC. Male coffee drinkers and non-smoking coffee drinkers were more likely to develop BC.

Bladder cancer is the most common cancer of the urinary tract and the ninth most common cancer among men, accounting for approximately 330,000 new cases and 130,000 deaths per year worldwide^1^. BC is a torturous disease which is hard to be cured and easy to relapse^2^. Additionally, it was reported that the cost per patient of BC from diagnosis to death was the highest of all cancers, ranging from US$96,000–187,000 due to long-term survival and the need for lifelong routine monitoring and treatment^3^. Moreover, the incidence of BC will probably increase in the next decades since the world population is increasing and the problem of the ageing is getting worse in the world. However, feasible measures for the prevention of BC remain limited due to the unknown cause of BC. Therefore, more etiology researches of BC should be conducted to provide theoretical basis for the prevention of BC.

Coffee is a popular drink worldwide, especially among western countries, which also possess high incidence of BC^1^,^4^. Researches on coffee-induced BC mechanisms had reported that several compounds in coffee, including caffeine, polycyclic aromatic hydrocarbons (PAHs), and nitrosamines could increase the risk of BC^5^,^6^. However, chemicals in coffee like diterpenes cafestol and kahweol had been found to reduce the chance of having BC^7^,^8^,^9^. Since the early 1970s, coffee consumption had been considered to be related with the risk of BC by a case-control study^10^ conducted by Cole. Several studies had been published thereafter, with inconsistent results reported^11^,^12^,^13^,^14^,^15^. Recently, a pooled analysis of the studies conducted in Europe showed non-smokers who were heavy coffee drinkers might have a small excess risk of BC^16^.

In order to clarify the association between coffee consumption and the risk of BC and provide theoretical basis for the prevention of BC, we conducted a meta-analysis of all published case-control and cohort studies.

## Results

### Study characteristics

**Figure 1:**
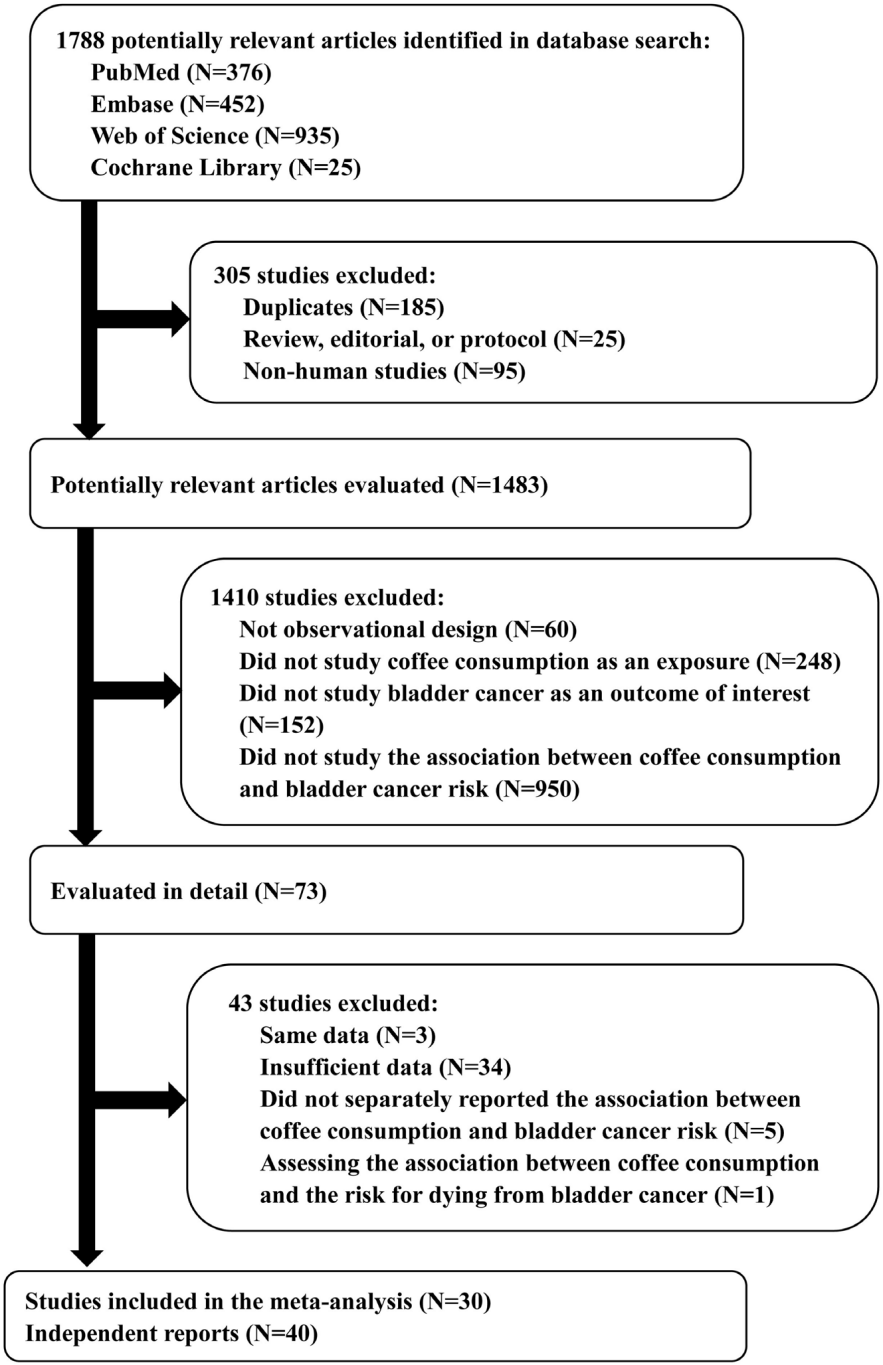
Flow chart showing the relevant observational studies of coffee consumption in relation to bladder cancer.

The study identification and selection progression was summarized in Figure 1. Totally, we identified 25 case-control studies^10^,^11^,^12^,^13^,^14^,^15^,^17^,^18^,^19^,^20^,^21^,^22^,^23^,^24^,^25^,^26^,^27^,^28^,^29^,^30^,^31^,^32^,^33^,^34^,^35^ including 15,419 cases and 23,585 controls, and 5 prospective cohort studies^36^,^37^,^38^,^39^,^40^ including 753 cases and 236,343 participants in our meta-analysis. Among the 25 case-control studies, 9 studies reported 2 separate outcomes stratified by gender (male and female). As for the 5 cohort studies, 1 study reported 2 separate outcomes stratified by gender (male and female). Thus, there were 40 independent reports included in this meta-analysis. General characteristics of the included studies had been shown in Supplementary Table S1 and Table S2. Among these studies, 14 were conducted in America, 5 in Asia and 22 in Europe. Besides, 17 studies included both male and female, 12 studies male only and 11 studies female only. According to the year of publication, 26 studies were published before 2000 (including 2000), while the remaining 14 studies were published after 2000. Among the 34 case-control studies, data of the types of control were also extracted, revealing that 20 studies were hospital-based, 12 studies were population-based and 2 studies were mixed with population-based and hospital-based controls. The estimated quality of all included studies was in the range of 3–7 scores. The ratings had been reported in the Supplementary Table S3 and Table S4.

### Association between coffee consumption and bladder cancer risk

**Figure 2:**
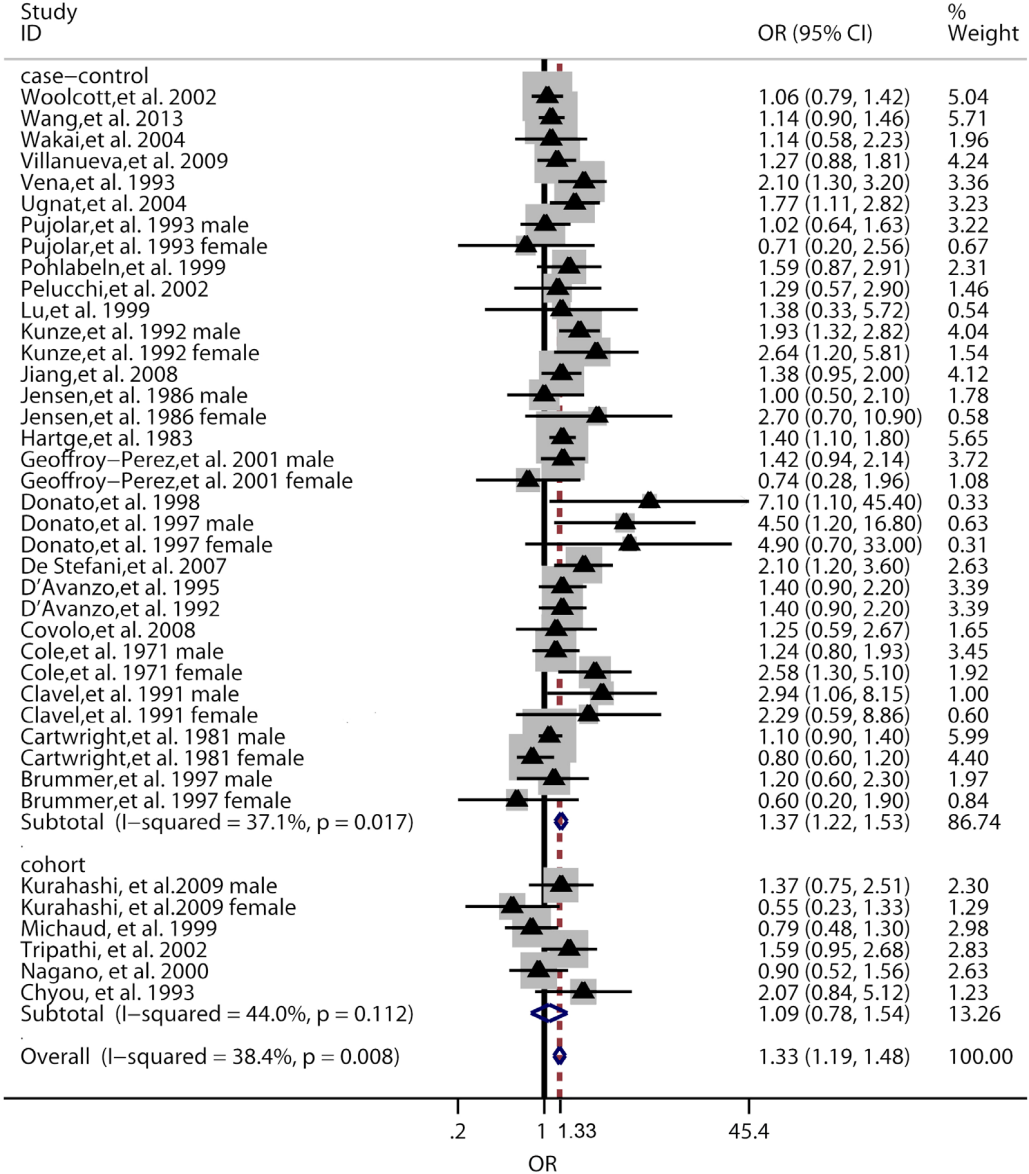
Pooled random effects OR and 95% CIs for the association of coffee consumption and bladder cancer. The triangles and horizontal lines correspond to the study-specific ORs and 95% CIs. The gray areas reflect the study-specific weight. The diamonds represent the pooled ORs and 95% CIs of each subgroup and overall population. The vertical solid line shows the OR of 1 and the vertical dashed line indicates the overall pooled OR of 1.33.

The results from the random-effect model combining the ORs for the risk of BC in relation to coffee consumption had been shown in Figure 2. A statistically significant association between the coffee intake and the risk of BC was found (OR = 1.33, 95% CI 1.19 to 1.48), and a moderate heterogeneity was detected (*P* = 0.008 for heterogeneity, I^2^ = 38.4%). For case-control studies, the combined OR was 1.37 (95% CI 1.22 to 1.53), with a moderate heterogeneity of *P* = 0.017 and I^2^ = 37.1%. For cohort studies, the combined OR was 1.10 (95% CI 0.78 to 1.54), with a moderate heterogeneity of *P* = 0.112 and I^2^ = 44.0%.

### Dose-response meta-analysis

**Figure 3:**
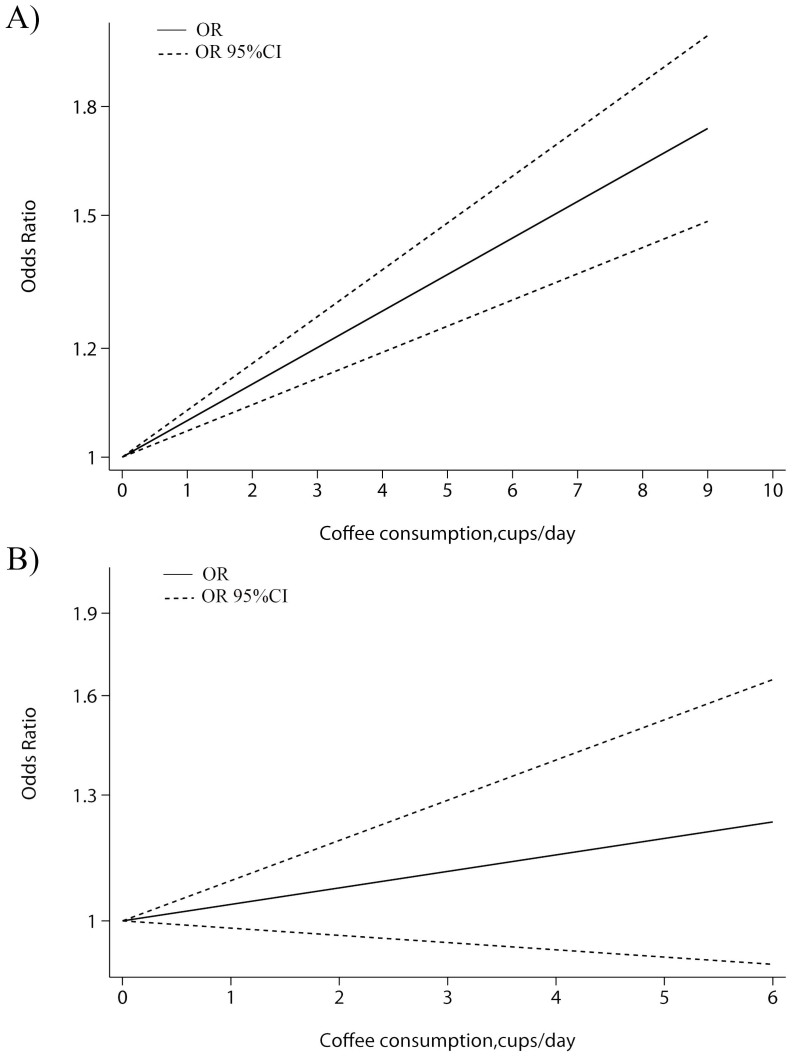
Dose-response relationship between coffee consumption and the risk of bladder cancer among A) case-control studies and B) cohort studies. The solid lines represent the linear trend. The dashed lines dashes represent the pointwise 95% confidence intervals for the linear trend.

Studies^10^,^11^,^12^,^25^ reporting less than three categories of coffee consumption were excluded in dose-response meta-analysis. Totally, 28 case-control studies and 6 cohort studies were included. The results had been shown in Figure 3. For case-control studies, we found no evidence of a nonlinearity association between coffee consumption and BC risk in case-control studies (*P* = 0.073 for nonlinearity). Thus, linear model had been applied among case-control studies. The summary relative risk of bladder cancer for an increase of one cup of coffee per day was 1.05 (95% CI 1.03 to 1.06; *P* = 0.001 for linear trend). As for cohort studies, a linear association was confirmed (*P* = 0.433 for nonlinearity). The summary relative risk of bladder cancer for an increase of one cup of coffee per day was 1.03 (95% CI 0.99 to 1.06; *P* = 0.001 for linear trend).

### Subgroup analyses

**Figure 4:**
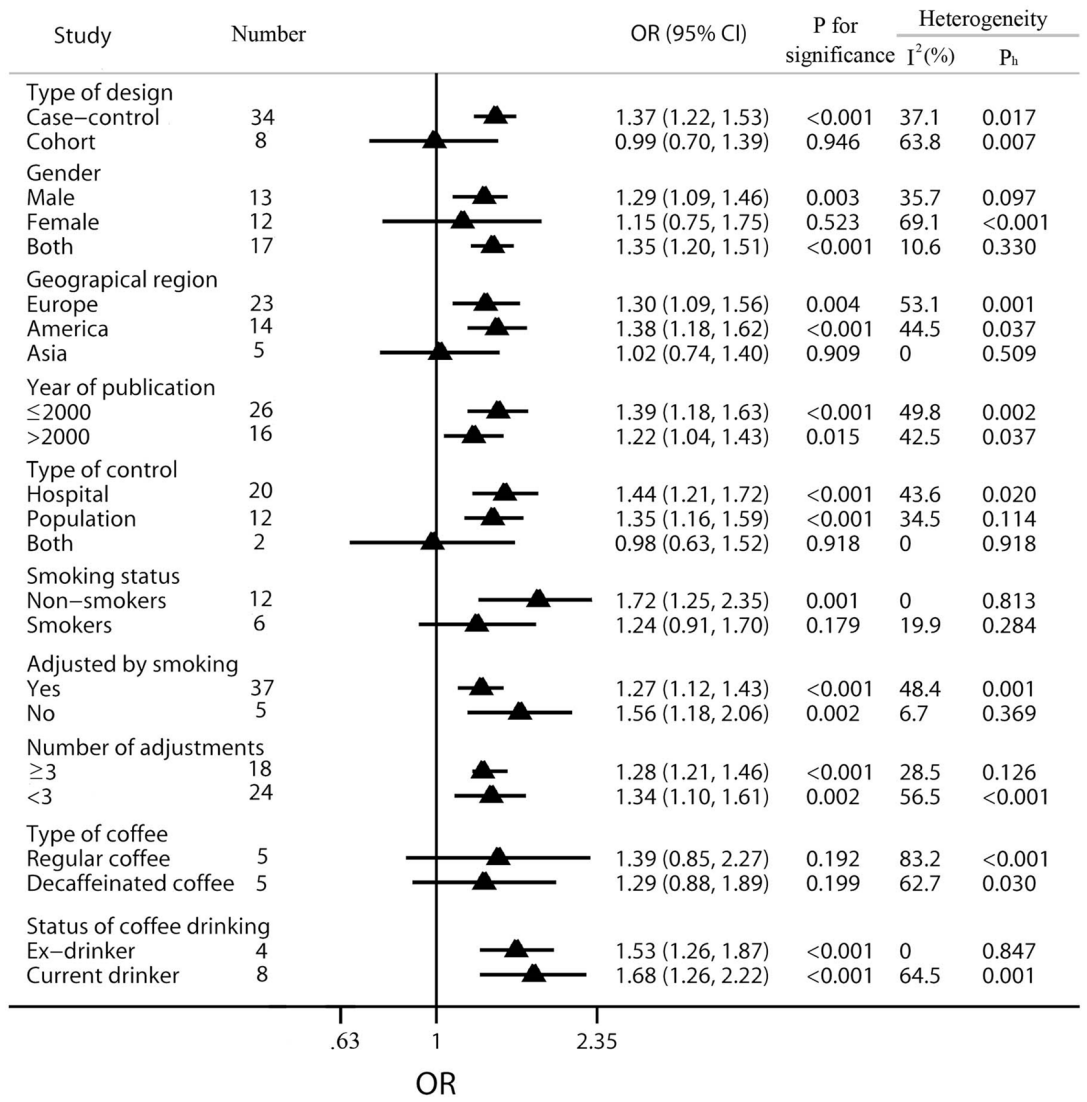
Subgroup analysis of OR of bladder cancer according to coffee consumption. The triangles and horizontal lines correspond to the subgroup-specific ORs and 95% CIs. The vertical solid line shows the OR of 1. “P_h_” represents the *P* value for heterogeneity from Q-test. Especially, the subgroup of type of control includes only the case-control studies, and “Both” indicates the study contains both hospital-based and population-based controls. For the subgroup stratified by the number of adjustments, “≥3” indicates the study should be adjusted at least three of the following factors: BMI, age, gender, smoking and other fluid intake.

Results of subgroup analyses had been shown in Figure 4. To assess the potential effects of specific study characteristics on the association between coffee consumption and BC risk, we pooled the ORs and 95% CIs from the subgroups of study design, gender, geographical region, year of publication, type of control (for case-control studies), smoking status and adjustments. In most subgroups, coffee consumption was associated with an increased risk of BC. In the subgroup of study design, a significantly increased risk was observed in the case-control studies (OR = 1.37, 95% CI: 1.22 to 1.53), while a non-significant association was found within the cohort studies (OR = 1.10, 95% CI: 0.78 to 1.54), with a *P* value for test of 0.603. When stratified by gender, the studies on male and both gender had shown a significantly increased association (male: OR = 1.31, 95% CI: 1.08 to 1.59, both: OR = 1.35, 95% CI: 1.20 to 1.51). However, the association among female group was lack of statistical significance (OR = 1.30, 95% CI: 0.87 to 1.96), with the *P* value of 0.204. According to geographical region, positive relations were detected in Europe (OR = 1.36, 95% CI: 1.15 to 1.62) and America (OR = 1.38, 95% CI: 1.18 to 1.62), but non-association was noted in Asia (OR = 1.02, 95% CI: 0.74 to 1.40). Moreover, in order to avoid residual confounding variables by smoking, we also conducted the subgroup analyses of whether the pooled ORs were adjusted for smoking or not and extracted 18 independent reports from the included 40 studies to evaluate the difference of the studied association between non-smokers and smokers. The results had shown that there was a greater increased risk among the smoking non-adjusted group (OR = 1.56, 95% CI: 1.18 to 2.06) than the smoking adjusted group (OR = 1.30, 95% CI: 1.16 to 1.46). Interestingly, we found a moderate increased risk in the non-smokers group (OR = 1.72, 95% CI: 1.25 to 2.35), but no significance in the smokers group (OR = 1.24, 95% CI: 0.91 to 1.70). Besides, high coffee consumption was found to be associated with BC risk among the subgroups of publication year (≤2000 or >2000) and adjustments (≥3 or <3). In order to further explore the potential association between different aspects of coffee consumption and the risk of BC, subgroup analyses of type of coffee and coffee drinking status had been conducted. The pooled OR for regular coffee group was 1.39 (95% CI 0.85 to 2.27), while the combined OR for decaffeinated group was 1.29 (95% CI 0.88 to 1.89). When stratified by coffee drinking status, significantly increased associations were found in both groups of ex-coffee drinker (OR = 1.53, 95% CI 1.26 to 1.87) and current coffee drinker (OR = 1.68, 95% CI 1.26 to 2.22).

### Sensitivity analyses

**Figure 5:**
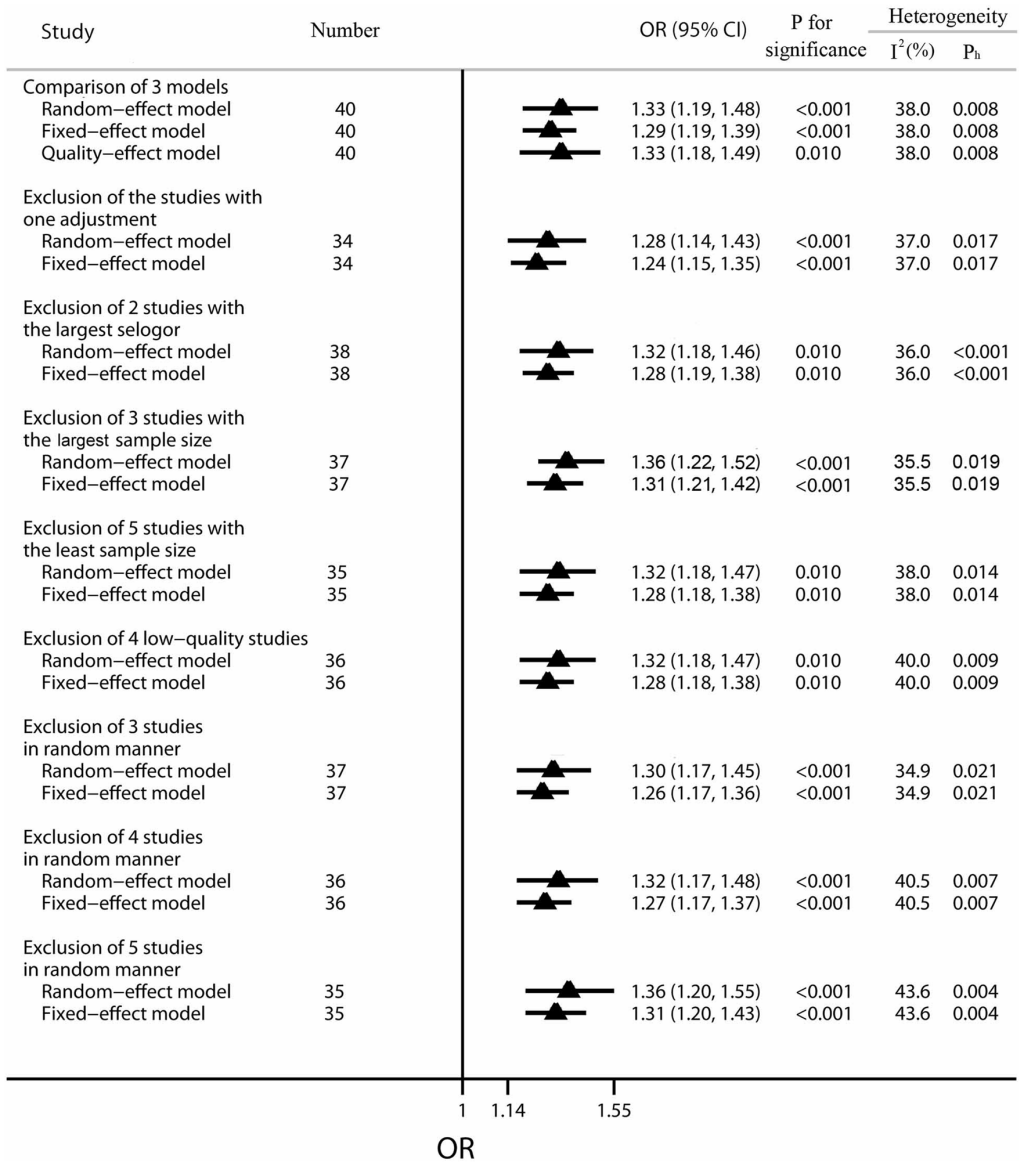
Results of sensitivity analysis on the association between coffee consumption and the risk of bladder cancer. The triangles and horizontal lines represent the corresponding ORs and 95% CIs. The vertical solid line shows the OR of 1. “P_h_” represents the *P* value for heterogeneity from Q-test. Especially, “studies with one adjustment” contains Tripathi, et al. 2002, Kunze, et al. 1992 male, Kunze, et al. 1992 female, Jensen, et al. 1986 male, Jensen, et al. 1986 female and Pohlabeln, et al. 1999; “2 studies possessing the largest selogor” represents the two studies with the largest standard error of OR (Donato, et al. 1998 and Donato, et al. 1997 female); “3 studies with the largest sample size” represents Kurahashi, et al.2009 female, Kurahashi, et al.2009 male and Michaud, et al. 1999; “5 studies with the least sample size” includes Clavel, et al. 1991 female, Pujolar, et al. 1993 female, Cole, et al. 1971 female, Geoffroy-Perez, et al. 2001 female and Donato, et al. 1997 female; “ 4 low-quality studies” indicates Pelucchi, et al. 2002, Donato, et al. 1997 male, Covolo, et al. 2008 and Donato, et al. 1997 female.

Sensitivity analyses were performed to evaluate the sensitivity of our conclusions about the overall effect of coffee consumption on the risk of BC. Firstly, the results of fixed-effect model, random-effect model and quality-effect model (Supplementary Figure S1) were compared to each other, and the results were robust (fixed-effects model pooled OR = 1.29, 95% CI 1.19 to 1.39; random-effects model pooled OR = 1.33, 95% CI 1.19 to 1.48; quality-effect model pooled OR = 1.33, 95% CI 1.18 to 1.49). Secondly, the leave-one-out analysis was performed by omitting one study in turn. The positive association was not drastically changed in the leave-one-out analysis, with pooled ORs ranging from 1.27 (95% CI 1.09 to 1.48) to 1.32 (95% CI 1.13 to 1.54). The results of the leave-one-out analysis had been displayed in Supplementary Figure S2. Thirdly, specific studies were excluded to further evaluate the reliability and stability of our conclusions. Exclusion of the studies with one adjustment, exclusion of the two studies possessing the largest standard error of effect estimates, exclusion of 3 studies with the largest sample size, exclusion of 5 studies with the least sample size and exclusion of 4 low-quality studies had been taken into account. Besides, we also omitted 3, 4 and 5 studies in random manner for further study. All the analyses had been performed applying both fixed-effect and random-effect models and the results were clearly shown in Figure 5.

### Publication bias

**Figure 6:**
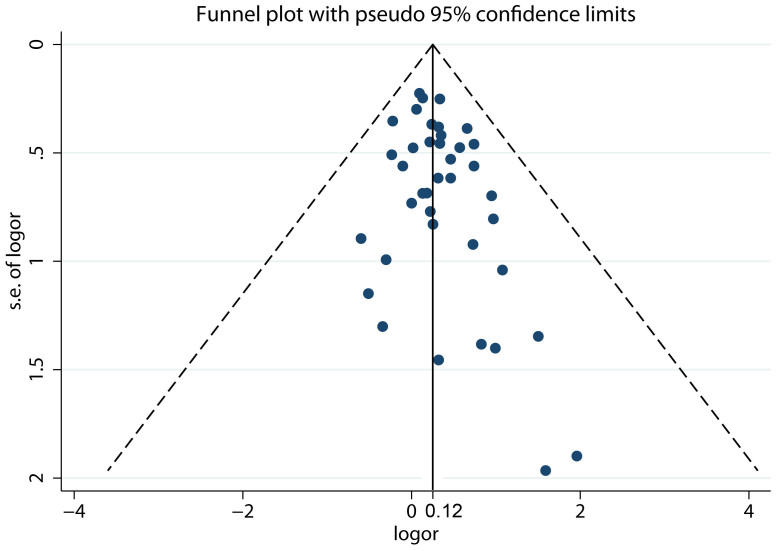
Funnel plot for studies of coffee consumption in relation to bladder cancer risk. The vertical solid line represents the summary effect estimates, and the dotted lines are pseudo 95% CIs.

Visual inspection of the funnel plot showed little asymmetry (Figure 6). The Egger test and Begg test did not suggest evidence of publication bias (Egger, *P* = 0.051; Begg, *P* = 0.139). Thus, no publication bias was observed in this study.

## Discussion

Drinking coffee can both facilitate and inhibit the occurrence and the development of BC in several mechanisms^21^,^22^,^29^. On the one side, coffee is a complex drink that contains many chemical compounds, including known or possible bladder carcinogens such as PAHs, nitrosamines and heterocyclic amines^5^. Besides, caffeine, mainly from coffee intake, has been demonstrated to affect DNA repair through the modification of the apoptotic response and perturbation of the cell cycle checkpoint integrity^6^. Moreover, the lethality of DNA-damaging agents, such as ionizing radiation or alkylating agents, is often potentiated when human cells are treated with caffeine^41^. However, on the other side, it should be noted that special compounds in coffee, including diterpenes cafestol and kahweol, polyphenols, caffeic acid and chlorogeni acid, can produce biological effects compatible with anti-carcinogenic properties to inhibit the occurrence and development of cancer^7^,^8^,^9^,^42^. In a research published in 2014, a hypothesis had been given that an increase of fluid intake would lead to the expansion of the bladder wall which would facilitate the direct actions of the carcinogens to deeper layer of the bladder urothelium^43^. However, meanwhile, a higher level of fluid consumption could increase urination frequency and reduce the exposure time of the carcinogens in turn, which can prevent the occurrence of cancer.

Potential bias is the major challenge for the meta-analysis of observational studies^44^. In the subgroup analyses of the type of control, the group of population-based controls (OR = 1.35, 95% CI: 1.16 to 1.59) had indicated a lower risk than the group of hospital-based controls (OR = 1.44, 95% CI: 1.21 to 1.72). This difference should be due to the various recall bias for the two types of controls. When considering the coffee is not good for health, cases and hospital-based controls might overstate their actual coffee consumption, while most of the population-based controls would properly evaluate their coffee intake. Thus, results from the population-based controls was thought to be more reliable. Besides, we also assessed the selection bias and investigation bias through the process of quality assessment (Supplementary Table S3 and Table S4).

In most cases, coffee drinkers tended to smoke, lack enough exercises and possess high body mass index (BMI), which had been reported to be the potential risk factors of the BC^45^,^46^,^47^. Owing to the correlations, the effect of coffee will be magnified in the presence of confounders. Therefore, adjustments should be conducted to reduce the potential confounding effect to get a more reliable conclusion. In the present meta-analysis, only studies with adjusted estimates were extracted. Influence of the potential confounding factors including smoking and BMI had been studied, and the result had shown that group with 3 or more adjustments (OR = 1.29, 95% CI: 1.11 to 1.49) exhibited a lower risk than the group with less than 3 adjustments (OR = 1.39, 95% CI: 1.18 to 1.64). This result had further confirmed the reliability of our findings, because the association remained unchanged even after the adjustments of the major confounding factors. Interestingly, there was a distinct difference in findings between studies published at or before 2000 (OR = 1.39, 95% CI: 1.18 to 1.63) and after 2000 (OR = 1.27, 95% CI: 1.12 to 1.43). The difference might result from better design of the studies published after 2000, which adjusted with more confounding factors, including BMI, tea consumption, physical activity and so on.

In particular, the interaction between coffee and smoking had been studied with more details within the present meta-analysis. Study between smokers and non-smokers had revealed that the risk of the BC among non-smokers (OR = 1.72, 95% CI: 1.25 to 2.35) was higher than smokers (OR = 1.24, 95% CI: 0.91 to 1.70). The observed outcome could be partly explained by coffee-induced BC mechanisms researches. It was reported that caffeine was the substrate of cytochrome P450 (CYP) enzymes in the liver, such as CYP1A2 or NAT2, and these enzymes might increase the metabolic activation of carcinogens like polycyclic aromatic hydrocarbons in cigarette smoking^6^. In the presence of smoke compounds, metabolism of caffeine could be faster^48^. Besides, experimental studies reported caffeine metabolism was induced by approximately 60 to 70% by cigarette smoke^49^. Thus, the adverse effects of coffee and caffeine may be more clearly expressed among never or former smokers and caffeine may modify the increased BC risk caused by smoking.

Bladder cancer is a complex disease resulting from interactions between genetic factors and environment^50^. The slow-acetylator phenotype of N-acetyltransferase and the polymorphisms of cytochrome P4501A2 has been found to be associated with a higher risk of BC within some epidemiological studies^51^. Recently, it was reported that the distribution of phenotypes of N-acetyltransferase varied between Asian and Caucasian population^37^,^52^, which could partly explain the different findings between Europe (OR = 1.36, 95% CI: 1.15 to 1.62) and America (OR = 1.38, 95% CI: 1.18 to 1.62), and Asia (OR = 1.02, 95% CI: 0.74 to 1.40). However, it should be noted that there were only 5 Asian studies included in our meta-analysis. More studies should be conducted in Asian countries to reveal the effect of genetic phenotypes in the relationship between coffee intake and BC.

There were several limitations in the present meta-analysis. First, we did not search for unpublished studies or original data, thus publication bias might be inevitable, even though no significant evidence of publication bias was observed. Second, categories of coffee consumption varied from studies, which might be responsible for the heterogeneity among studies in this analysis. Third, owing to the small number of Asian studies, the selection bias was unavoidable and the association among different countries remained unclear. Besides, due to the lack of relevant studies, important aspects of coffee drinking, including duration of coffee consumption, type of coffee and the status of coffee drinking, had not been studied enough. Furthermore, major risk factors including high risk occupations, disinfection byproducts, arsenic in the drinking water and genotype for the major metabolic enzymes were hard to be avoided.

In summary, the present meta-analysis, consisting of 34 case-control studies and 6 cohort studies, had suggested a significantly increased risk between coffee consumption and the risk of BC. Subgroup analyses indicated that male drinkers and non-smokers with high coffee consumption were more likely to develop BC. However, caution is needed in interpreting the findings from our meta-analysis because of the inevitable heterogeneity. Further well-designed large-scaled studies are warranted to provide more definitive conclusions.

## Methods

### Search strategy

In this paper, we conducted a literature search of PubMed (Medline), Embase, Web of Science and Cochrane Library from January 1966 through August 2014 for observational studies examining the association between coffee consumption and risk of BC. Search terms including “coffee”, “caffeine”, “drink”, “beverage” or “fluid” combined with “bladder cancer”, “bladder tumor”, “bladder neoplasm” or “bladder carcinoma” had been applied in the database search. Besides, reference lists from retrieved articles were also reviewed. Only articles published in English were considered. We followed the standard criteria for conducting meta-analyses of observational studies and reporting the results^53^.

### Inclusion criteria

Studies meeting the following criteria were included in the meta-analysis: (1) the study design was observational, (2) the exposure of interest was coffee consumption, (3) the outcome of interest was bladder cancer, (4) the study reported adjusted risk estimates (relative risks or hazard risks or odds risks) with their 95% CI for the association between coffee consumption and BC or provided sufficient information to allow their calculations, (5) the study provided the frequency of every category of coffee consumption. Additionally, we excluded reviews, editorials, non-human studies, and letters without sufficient data. When multiple reports based on the same study were published, only the most recent or complete report would be used.

### Data extraction

Eligibility evaluation and data extraction were carried out independently by two reviewers (W.W. and Y.T.) according to the guidelines for meta-analysis^53^. Discrepancies were adjudicated by discussions with a third reviewer (Q.L.). The following information were extracted from all the identified studies: name of first author, year of publication, country where the study was performed, type of study design, characteristics of the study population, duration of follow-up (for cohort study), number of cases or controls (participants and person-years for cohort studies), variables adjusted for in the analysis, as well as multivariate adjusted ORs (HRs or RRs) and 95% CIs for each category of coffee consumption. Only risk estimates adjusted with covariates were extracted. For studies that applied different models for the calculation of estimate risks, we chose the results adjusted with more potential confounders. In particular, we extracted ORs (RRs or HRs) adjusted for smoking when they were available, as smoking was a major confounder for the possible relationship between coffee consumption and risk of BC. Besides, ORs (RRs or HRs) among non-smokers and smokers were also extracted for further studying the confounding effect of smoking.

### Quality assessment

Two reviewers (W.W. and Y.T.) independently performed the quality assessment using the Newcastle-Ottawa Scale^54^ (for the cohort and case–control study), which is a nine-point scale allocating points based on the selection process of studies (0–4 points), the comparability of studies (0–2 points) and the identification of the exposure and the outcomes of study participants (0–3 points). The quality of the articles was first evaluated according to the established questions, which were scored according to the follows: 1 point if the item was considered in the study, 0 point if the item was not considered or it was impossible to determine whether it was considered or not. We assigned scores of 0–3, 4–6 and 7–9 for low, moderate and high quality of studies, respectively.

### Statistical analysis

The statistical analyses for the overall association between coffee consumption and BC risk were based on comparisons of the highest intake category of coffee consumption with the reference. In most cases, the reference refer to the lowest consumption of coffee, including the non-drinkers. Among the 30 identified studies, 10 studies^10^,^11^,^13^,^15^,^22^,^26^,^28^,^29^,^31^,^37^ did not offer pooled risk estimates for the whole research population but they provided separate estimates for each group by gender. Under this circumstance, each of these studies would be considered as two independent reports. Thus, 40 independent reports were included in our meta-analysis. It was reported that when the outcome was rare, ORs, HRs and RRs would provide similar estimates of risk^55^. In this work, ORs were chosen as a common measure of the association between coffee consumption and BC risk.

The median or mean coffee consumption in each category was used as the corresponding dose of consumption. Different measurements of coffee consumption in the original studies were converted into approximate cups/day. We defined 240 ml or 8 oz of coffee equal to one cup. When the median or mean consumption per category was not reported in the article, we assigned the midpoint of the upper and lower boundaries in each category as the average consumption. If the highest category was open-ended, we set the midpoint of the category at 1.5 times the lower boundary. When the lowest category was open-ended, the lower boundary was set to zero.

In the dose-response analysis, the number of cases and the total number of objects were used as covariates among case-control studies, while the number of cases and person-years were applied among cohort studies. Thus, analyses had been conducted for case-control studies and cohort studies, respectively. We performed the dose–response analysis to examine a potential nonlinear relationship. This was done by modeling coffee consumption using restricted cubic splines with 3 knots at fixed percentiles 10%, 50%, and 95% of the distribution. A *P* value for nonlinearity would be calculated by testing the null hypothesis that the coefficient of the second spline was equal to zero^56^.

The heterogeneity among studies was estimated by the Cochran Q test and I^2^ statistic^44^. Heterogeneity was confirmed with a significance level of *P* ≤ 0.10. For the I^2^ metric, low, moderate, and high I^2^ values were considered to be 25%, 50%, and 75%, respectively^44^. Fixed effect model would be applied when heterogeneity was negligible, otherwise random effect model would be used^57^. Besides, quality-effect model^58^ based on quality scores was employed and the results were compared with those from random-effect model and fixed-effect model.

To explore the potential heterogeneity among studies, subgroup analyses were conducted according to study design, gender, geographical region, publication year, adjustments. Besides, subgroup analyses of type of coffee and coffee drinking status had been conducted to further explore the potential association between different aspects of coffee consumption and the risk of BC. Sensitivity analyses were employed to find potential origins of heterogeneity and to examine the influence of various exclusions on the combined OR. Publication bias was assessed through the visual inspection of funnel plots and with tests of Begg rank correlation^59^ and Egger regression asymmetry^60^. *P* < 0.05 was considered to be representative of a significant statistical publication bias. Forest plots were applied to assess the overall association between coffee consumption and BC.

In addition to quality-effect modeling (conducted with MetaXL version 2.2 software), all statistical analyses were performed with STATA version 12.0 software (Stata Corporation, College Station, Texas, United States). All reported probabilities (*P* values) were two-sided, with *P* < 0.05 considered statistically significant.

## Author Contributions

All authors contributed significantly to this work. W.W., Y.T. and Q.L. designed the research study; W.W., Y.T., Q.Z., G.Y. and X.W. performed the research study and collected the data; W.W., Y.T. and Q.Z. analyzed the data; W.W., Y.T., Q.L. wrote the paper; Q.Z., G.Y. and X.W. prepared Figures 1–6, Figure S1–S2 and Supplemental Table S1–S4. All authors reviewed the manuscript. In addition, all authors approved the final draft.

## Supplementary Material

Supplementary Information Supplementary Information
