# Supplementary material for: Coffee consumption and bladder cancer: a meta-analysis of observational studies
Source: Sci Rep. 2015 Mar 12;5:9051. doi: 10.1038/srep09051 (PMC4356958; doi:10.1038/srep09051)
Supplement: Supplementary Information [file srep09051-s1.pdf]

Supplementary Information

Coffee consumption and bladder cancer: a meta-analysis of observational studies

Weixiang Wu[1,2]; Yeqing Tong[1,3]; Qiang Zhao[2]; Guangxia Yu[2]; Xiaoyun Wei[2]; Qing Lu[2,*]

[1] These authors contributed equally to this work

[2]Key Laboratory of Environment and Health, Ministry of Education & Ministry of Environmental Protection, and State Key Laboratory of Environmental Health (Incubating), School of Public Health, Tongji Medical College, Huazhong University of Science and Technology

[3]Hubei provincial center for disease control and prevention

[*] Corresponding author:

Qing Lu; E-mail: qi_weiliao@126.com

Address: School of Public Health, Tongji Medical College, Huazhong University of Science and Technology, #13 Hangkong Road, Wuhan, Hubei, 430030, China;

Phone: +86-27-83625912;

Fax: +86-27-83657765.

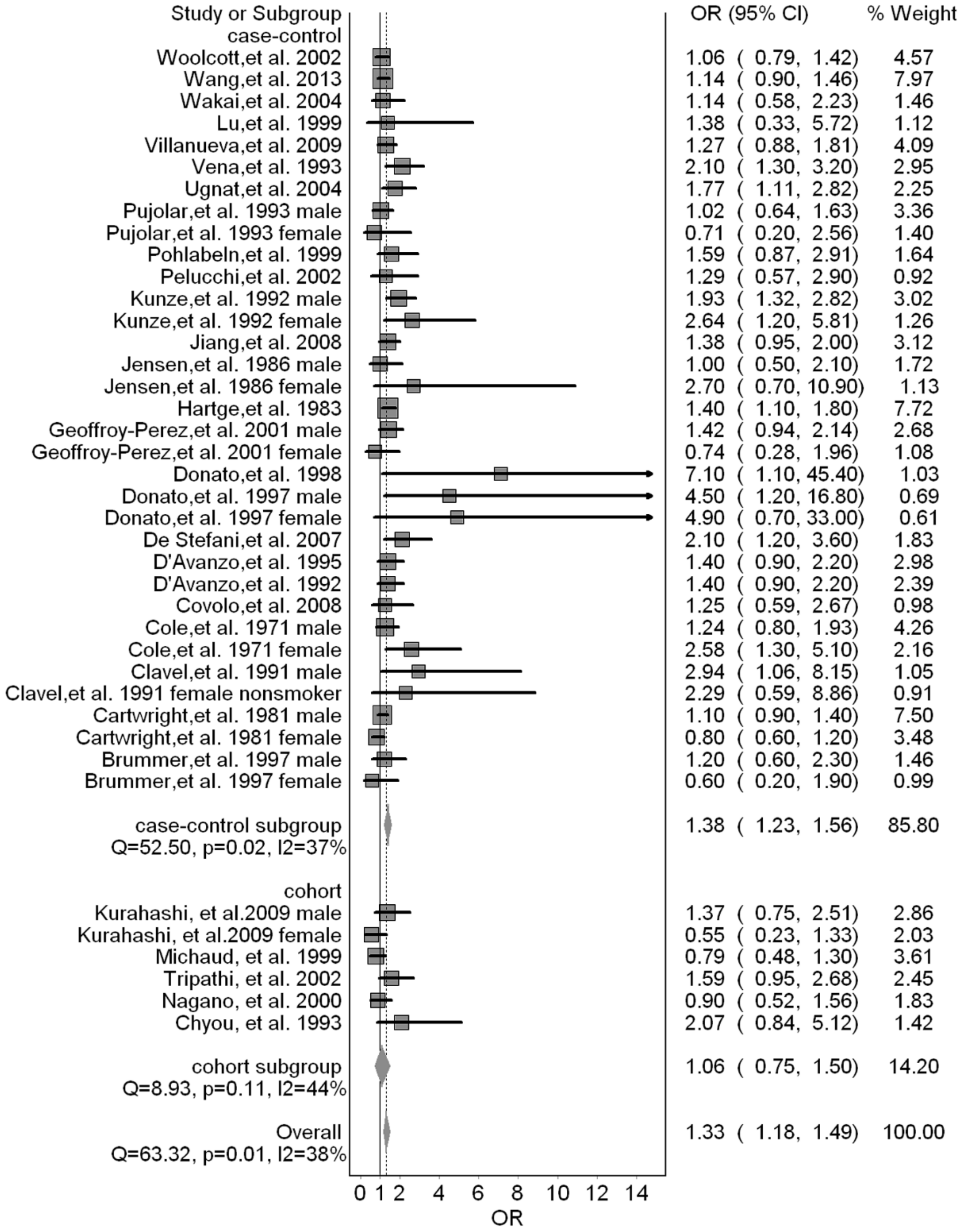

Supplementary Figure S1. Pooled quality effects OR and 95% CI for the association between coffee consumption and the risk of bladder cancer. The horizontal lines correspond to the study-specific ORs and 95% CIs. The gray squares reflect the study-specific weight. The diamonds represent the pooled ORs and 95% CIs of each subgroup and overall population. The vertical solid line shows the OR of 1 and the vertical dashed line indicates the overall pooled OR of 1.33.

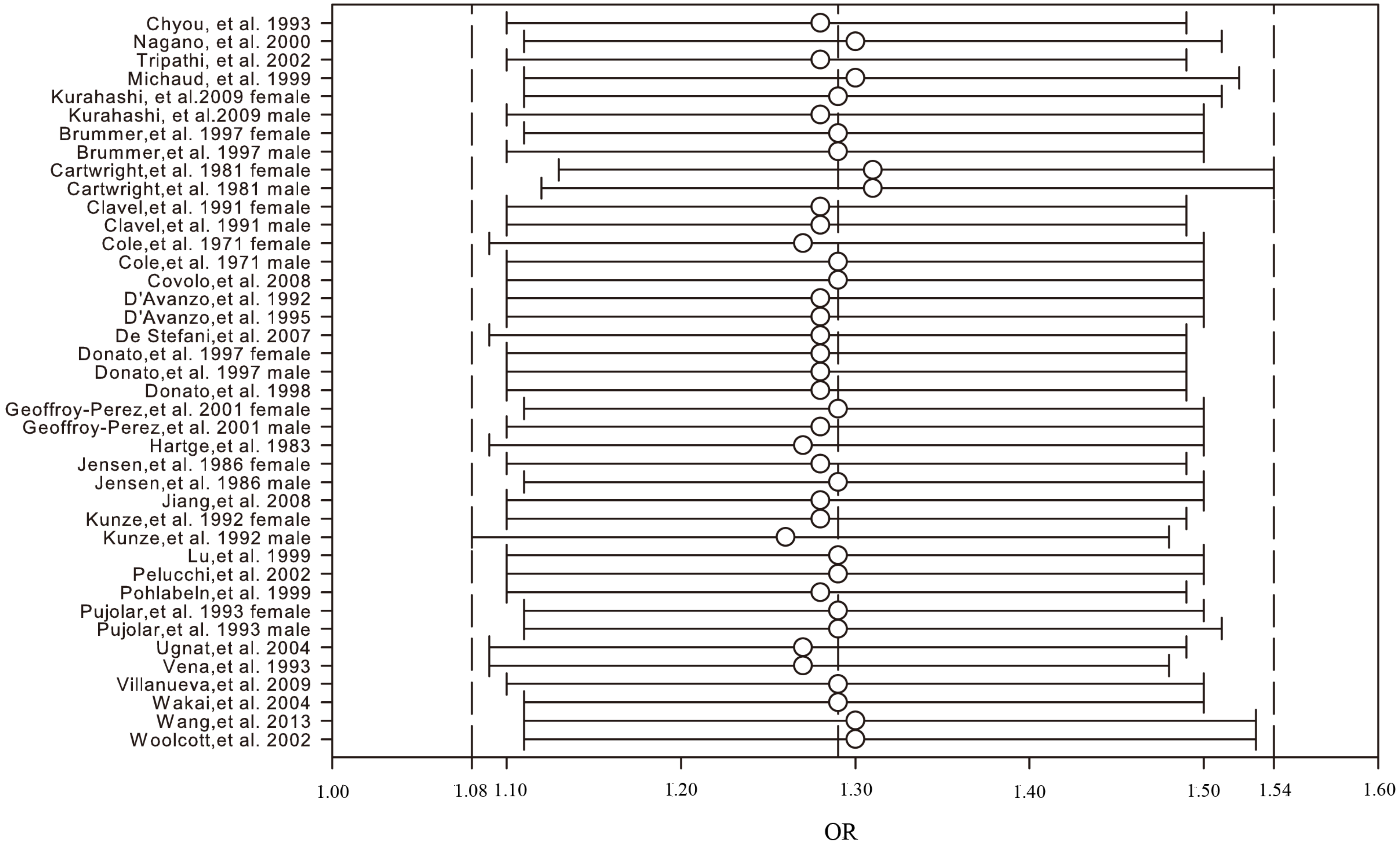

Supplementary Figure S2. Results of sensitivity analysis using leave-one-out method. The circles and the horizontal lines represent the ORs and 95% CIs after omitting studies in turn. The vertical dashed lines show the OR of 1.08 and 1.54.

Supplementary Table S1. Characteristics of case-control studies on coffee consumption in relation to bladder cancer

Study	Year	Type of control	Country	Gender[a]	Case/control	coffee consumption	OR(95% CI)[b]	Adjustments
Woolcott,et al.	2002	population	Canada	F/M	150/436	<1cups/day	1.00(reference)	Age, sex education level, current smoking , cumulative smoking , and intake of energy , calcium , fibre and beer
					320/734	1-2cups/day	1.03(0.81-1.32)	
					278/661	2-3cups/day	0.88(0.68-1.13)	
					165/271	⩾5cups/day	1.06(0.79-1.42)	
Wang,et al.	2013	population	USA	F/M	259/155	Never	1.00(reference)	Age, sex, ethnicity, energy intake, and smoking
					375/271	0.1-1.9cups/day	1.13(0.87-1.47)	
					665/581	2+cups/day	1.14(0.90-1.46)	
Wakai,et al.	2004	hospital	Japan	F/M	26/145	Almost never	1.00(reference)	Age, sex, year of first visit, and cumulative consumption of cigarettes
					23/123	Occasionally	0.93(0.52-1.66)	
					28/163	1cups/day	0.82(0.47-1.44)	
					26/113	2cups/day	1.07(0.59-1.94)	
					21/76	⩾3cups/day	1.14(0.58-2.23)	
Villanueva,et al.	2009	hospital	Spain	F/M	120/166	Never	1.00(reference)	Smoking, Age, gender and area
					1016/972	Ever	1.25(0.95-1.64)	
					336/352	1cups/day	1.24(0.92-1.66)	
					303/321	2cups/day	1.11(0.82-1.51)	
					223/165	3cups/day	1.57(0.13-2.19)	
					154/134	4+cups/day	1.27(0.88-1.81)	
Vena,et al.	1993	population	USA	F/M	60/205	0-1cups/day	1.00(reference)	Age, education, cigarette smoking and other liquids , sodium, carotene and calories
					62/170	2cups/day	1.3(0.80-2.00)	
					114/270	3-4cups/day	1.6(1.10-2.30)	
					115/210	5+cups/day	2.1(1.30-3.20)	
Ugnat,et al.	2004	population	Canada	F/M	34/142	<1cups/month	1.00(reference)	Age, province, education, smoking ,coffee, and tea consumption
					89/263	>=1cups/month and ⩽1 cups/day	1.13(0.69-1.83)	
					214/400	2-3 cups/day	1.56(0.99-2.46)	
					210/282	⩾4cups/day	1.77(1.11-2.82)	
Pujolar,et al.	1993	mix	Spain	M	34/103	⩽1cups/week	1.00(reference)	
					138/326	2-7cups/week	0.99(0.63-1.57)	

					130/294	8-14cups/week	0.95(0.59-1.51)	Tabacoo consumption, status as smoker, occupation, consumption of artificial sweeteners, age and province of residence
					135/263	15+cups/week	1.02(0.64-1.63)	
Pujolar,et al.	1993	mix	Spain	F	5/10	≤1cups/week	1.00(reference)	
					17/37	2-7cups/week	1.02(0.29-3.58)	Tabacoo consumption, status as smoker, occupation, consumption of artificial sweeteners, age and province of residence
					24/42	8-14cups/week	1.14(0.34-3.85)	
					13/38	15+cups/week	0.71(0.20-2.56)	
Pohlabeln,et al.	1999	hospital	German	F/M	53/83	≤1cups/day	1.00(reference)	
					128/115	2-4cups/day	1.51(0.95-2.39)	Smoking
					58/41	≥5cups/day	1.59(0.87-2.91)	
Pelucchi,et al.	2002	hospital	Italy	F/M	16/43	Nondrinkers	1.00(reference)	
					94/255	Drinkers	1.65(0.82-3.33)	
					26/68	1cups/day	1.84(0.81-4.21)	Age, study center, education, BMI, coffee and alcohol consumption and cigarette smoking
					34/94	2cups/day	1.92(0.87-4.20)	
					34/93	≥3cups/day	1.29(0.57-2.90)	
Lu,et al.	1999	hospital	Taiwan	F/M	36/151	No	1.00(reference)	Age, sex, date of admission, family history, ethnicity and smoking status
					4/9	Yes	1.38(0.33-5.72)	
Kunze,et al.	1992	hospital	German	M	168/194	1~2cups/day	1.00(reference)	
					205/182	2-3cups/day	1.30(0.98-1.73)	Smoking status
					102/61	5+cups/day	1.93(1.32-2.82)	
Kunze,et al.	1992	hospital	German	F	47/62	1-2cups/day	1.00(reference)	
					60/49	2-3cups/day	1.62(0.95-2.76)	Smoking status
					24/12	5+cups/day	2.64(1.20-5.81)	
Jiang,et al.	2008	population	USA	F/M	129/190	0cups/day	1.00(reference)	
					49/64	<1cups/day	1.15(0.71-1.38)	
					501/588	1~2cups/day	1.04(0.78-1.38)	Education, use of NSAIDs, number of years as a hairdresser/barber, cigarette smoking status, duration of smoking and intensity of smoking
					467/414	3-4cups/day	1.21(0.89-1.64)	
					226/193	5-6cups/day	1.19(0.85-1.68)	
					210/137	≥7cups/day	1.38(0.95-2.00)	
Jensen,et al.	1986	population	Denmark	M	15/33	0ml/day	1.00(reference)	
					69/148	1-499ml/day	0.9(0.50-1.90)	Smoking
					90/204	500-999ml/day	0.8(0.40-1.60)	
					56/108	1000-1499ml/day	0.9(0.40-1.80)	

					50/84	1500+ml/day	1.0(0.50-2.10)	
Jensen,et al.	1986	population	Denmark	F	4/17	0ml/day	1.00(reference)	
					20/48	1-499ml/day	1.9(0.60-6.70)	
					33/85	500-999ml/day	1.2(0.40-3.50)	Smoking
					15/30	1000-1499ml/day	1.6(0.40-6.00)	
					13/14	1500+ml/day	2.7(0.70-10.90)	
Hartge,et al.	1983	population	USA	F/M	98/365	Never drank	1.00(reference)	Age, race, geographic area, and tobacco history
					2809/5289	Ever drank	1.4(1.10-1.80)	
Geoffroy-Perez,et al.	2001	hospital	France	M	83/127	≤1050(ml/week)	1.00(reference)	
					116/117	1051-2050(ml/week)	1.45(0.97-2.16)	
					133/124	2051-2400(ml/week)	1.54(1.04-2.28)	Age, center and place of residence and smoking(coffee cup meal 75ml)
					127/117	2401-2800(ml/week)	1.62(1.08-2.40)	
					134/121	>2800(ml/week)	1.42(0.94-2.14)	
Geoffroy-Perez,et al.	2001	hospital	France	F	20/26	≤1150(ml/week)	1.00(reference)	
					38/31	1151-2100(ml/week)	1.40(0.63-3.12)	Age, center and place of residence and smoking(coffee cup meal 75ml)
					28/24	2101-2600(ml/week)	1.25(0.53-2.98)	
					19/24	>2600(ml/week)	0.74(0.28-1.96)	
Donato,et al.	1998	hospital	Italy	F/M	15/48	Non-drinker	1.00(reference)	
					56/70	1-2cups/day	2.9(1.1-7.8)	Smoking status, age, sex, education and residence according to interview
					33/34	3-4cups/day	3.5(1.2-10.1)	
					5/3	5+cups/day	7.1(1.1-45.4)	
Donato,et al.	1997	hospital	Italy	M	7/72	Non-drinker	1.00(reference)	
					6/17	Ex-drinker	2.7(0.70-10.30)	
					122/309	Current drinker	2.6(1.10-6.10)	
					66/203	1-2cups/day	2.3(0.90-5.60)	Age, residence, education and date of interview
					44/89	3-4cups/day	2.8(1.10-7.40)	
					11/17	5+cups/day	4.5(1.20-16.80)	
Donato,et al.	1997	hospital	Italy	F	2/27	Non-drinker	1.00(reference)	
					0/8	Ex-drinker	NA	
					35/145	Current drinker	5.2(1.00-30.40)	Age, residence, education and date of interview
					27/98	1-2cups/day	4.3(0.80-23.90)	
					8/47	3+cups/day	4.9(0.70-33.00)	

De Stefani,et al.	2007	hospital	Uruguay	F/M	135/332	Never drinkers	1.00(reference)	Age, sex, residence, urban/rural status, education, family history of bladder cancer among first-degree relative, BMI, occupation, smoking status, years since quitting, number of cigarettes smoked per day, mate drinking, soft drink intake, milk intake, and tea drinking
					84/133	1-6cups/week	1.5(1.10-2.20)	
					36/36	7+cups/week	2.1(1.20-3.60)	
D'Avanzo,et al.	1995	hospital	Italy	F/M	62/98	0/day	1.00(reference)	Age, sex, area of residence
					229/255	1-2/day	1.3(0.9-2.0)	
					140/138	≥3/day	1.4(0.9-2.2)	
D'Avanzo,et al.	1992	hospital	Italy	F/M	71/135	0cups/day	1.00(reference)	Age, sex education, smoking habits, current smoker, alcohol drinking and exposure to occupation at risk
					126/212	1cups/day	1.2(0.80-1.70)	
					167/249	2cups/day	1.4(0.90-2.00)	
					109/149	3cups/day	1.5(1.00-2.20)	
					82/110	≥4cups/day	1.4(0.90-2.20)	
Covolo,et al.	2008	hospital	Italy	F/M	26/30	Non-drinkers	1.00(reference)	Age, education, PAHs and AA exposure and cumulative lifetime smoking
					125/150	1~3cups/day	0.76(0.41-1.41)	
					77/31	>3cups/day	1.25(0.59-2.67)	
Cole,et al.	1971	population	USA	M	29/32	Non-drinker	1.00(reference)	Age, cigarette-smoking and occupation
					316/316	Coffee-drinker	1.24(0.80-1.93)	
Cole,et al.	1971	population	USA	F	9/9	Non-drinker	1.00(reference)	Age, cigarette-smoking and occupation
					91/91	Coffee-drinker	2.58(1.30-5.10)	
Clavel,et al.	1991	hospital	France	M	12/20	0cups/day	1.00(reference)	Smoker status, age, hospital and place of residence
					488/511	1-4cups/day	1.24(0.56-2.74)	
					61/52	5-7cups/day	1.46(0.60-3.51)	
					27/10	>7cups/day	2.94(1.06-8.15)	
Clavel,et al.	1991	hospital	France	F	3/5	0cups/day	1.00(reference)	Age, hospital and residence
					7/11	1cups/day	0.99(0.34-2.93)	
					16/24	2cups/day	1.51(0.48-4.74)	
					13/16	3cups/day	2.29(0.59-8.86)	
					15/13	>3cups/day	NA	
					1/3	Unknown	NA	
Cartwright,et al.	1981	hospital	England	M	294/417	Never drink coffee	1.00(reference)	Type of case and cigarette smoking
					337/372	Coffee drinker	1.1(0.90-1.40)	
Cartwright,et al.	1981	hospital	England	F	81/114	Never drink coffee	1.00(reference)	Type of case and cigarette smoking
					129/157	Coffee drinker	0.8(0.60-1.20)	

Brummer,et al.	1997	population	USA	M	24/32	None	1.00(reference)	Age, country and smoking
					50/72	≤3cups/day	1.10(0.50-2.10)	
					77/60	>3-6cups/day	1.70(0.90-3.40)	
					51/56	>6cups/day	1.20(0.60-2.30)	
Brummer,et al.	1997	population	USA	F	11/24	None	1.00(reference)	Age, country and smoking
					21/79	≤3cups/day	0.5(0.20-1.20)	
					20/56	>3-6cups/day	0.5(0.20-1.30)	
					8/26	>6cups/day	0.6(0.20-1.90)	

[a] F is short for Female and M is short for Male. F/M means the study includes both female and male objects.

[b] OR is short for odds ratio, and CI is short for confidence interval. All the ORs are adjusted. NA means the data is not available.

Supplementary Table S2. Characteristics of cohort studies on coffee consumption in relation to bladder cancer

Study	Year of publication	Country	follow-up period	Gender[a]	Case/Person-years	Coffee consumption	OR(95% CI)[b]	Adjustments
Kurahashi, et al.	2009	Japan	15	M	50/185405	Almost none	1.00(reference)	Age, area, smoking status, alcohol drinking, green tea drinking
					52/183367	1-4 times/week	1.26(0.84-1.88)	
					43/157544	1-2 cups/day	1.53(0.98-2.37)	
					19/83713	⩾3 cups/day	1.37(0.75-2.51)	
Kurahashi, et al.	2009	Japan	15	F	19/226689	Almost none	1.00(reference)	Age, area, smoking status, alcohol drinking, green tea drinking
					15/207355	1-4 times/week	1.03(0.51-2.07)	
					8/270514	⩾1 cups/day	0.55(0.23-1.33)	
Michaud, et al.	1999	USA	10	M	75/145351	<1 cups/month	1.00(reference)	Geographic region, age, pack-years of smoking, current smoking status, energy intake and intake of fruits and vegetables
					56/101672	1 cup/month – 6 cups/week	0.97(0.68-1.37)	
					98/165995	1-3 cups/day	0.99(0.73-1.37)	
					23/48961	⩾4 cups/day	0.79(0.48-1.3)	
Tripathi, et al.	2002	USA	13	F	28/118194	Never or <1cup/month	1.00(reference)	Age
					19/79048	1 cup/month to 5-6 cups/week	1.01(0.56-1.79)	
					34/144297	1 cup/day to 2-3 cups/day	1.01(0.6-1.64)	
					29/80657	≥4 cups/day	1.59(0.95-2.68)	
Nagano, et al.	2000	Japan	14	F/M	25/74670	0 cup/day	1.00(reference)	Age, gender, radiation dose, smoking status, education level, BMI, calendar time
					32/134070	1-4 cups/day	0.73(0.43-1.25)	
					32/122971	⩾5 cups/day	0.9(0.52-1.56)	
Chyou, et al.	1993	USA	22	M	5/942	⩽1 cup/day	1.00(reference)	Age , smoking status
					5/253	1-4 cups/day	3.52(1.02-12.2)	
					86/6703	⩾5 cups/day	2.07(0.84-2.12)	

[a] F is short for Female and M is short for Male. F/M means the study includes both female and male objects.

[b] OR is short for odds ratio, and CI is short for confidence interval. In this meta-analysis, the ORs were chosen as a common measure of the association between coffee consumption and bladder cancer.

Supplementary Table S3. Quality assessment of the case-control studies on coffee consumption in relation to bladder cancer

	Selection				Comparability		Exposure			
	1	2	3	4	5A	5B	6	7	8	
study	Indicates cases independently validated	Cases are representative of population	community controls	controls have no history of bladder cancer	study controls for age	study controls for additional factor	ascertainment of exposure by blinded interview or record	same method of ascertainment used for cases and controls	nonresponse rate the same for cases and controls	Overall quality score
Pelucchi,et al. 2002	0	0	0	1	1	0	0	1	0	3
Donato,et al. 1997 male	0	0	0	0	1	1	0	1	0	3
Donato,et al. 1997 female	0	0	0	0	1	1	0	1	0	3
Covolo,et al. 2008	0	0	0	0	1	1	0	1	0	3
Woolcott,et al. 2002	0	0	1	0	1	1	0	1	0	4
Wakai,et al. 2004	0	0	0	1	1	1	0	1	0	4
Ugnat,et al. 2004	0	0	1	0	1	1	0	1	0	4
Pohlabeln,et al. 1999	0	0	0	1	1	1	0	1	0	4
Kunze,et al. 1992 male	0	0	0	1	1	1	0	1	0	4
Kunze,et al. 1992 female	0	0	0	1	1	1	0	1	0	4
Jiang,et al. 2008	0	0	1	0	1	1	0	1	0	4
Geoffroy-Perez,et al. 2001 male	0	0	0	1	1	1	0	1	0	4
Geoffroy-Perez,et al. 2001 female	0	0	0	1	1	1	0	1	0	4
De Stefani,et al. 2007	0	0	0	1	1	1	0	0	1	4
D'Avanzo,et al. 1992	0	0	0	1	1	0	0	1	1	4
Clavel,et al. 1991 male	0	0	0	1	1	1	0	1	0	4
Clavel,et al. 1991 female nonsmoker	0	0	0	1	1	1	0	1	0	4
Cartwright,et al. 1981 male	0	1	0	1	1	1	0	0	0	4
Cartwright,et al. 1981 female	0	1	0	1	1	1	0	0	0	4
Brummer,et al. 1997 male	0	0	1	0	1	1	0	1	0	4

Brummer,et al. 1997 female	0	0	1	0	1	1	0	1	0	4
Wang,et al. 2013	0	0	1	1	1	1	0	1	0	5
Villanueva,et al. 2009	1	0	0	1	1	1	0	1	0	5
Vena,et al. 1993	0	1	1	0	1	1	0	1	0	5
Lu,et al. 1999	0	1	0	1	1	1	0	1	0	5
Jensen,et al. 1986 male	0	1	1	0	1	1	0	1	0	5
Jensen,et al. 1986 female	0	1	1	0	1	1	0	1	0	5
Hartge,et al. 1983	0	1	1	0	1	1	0	1	0	5
Donato,et al. 1998	0	1	0	0	1	1	1	1	0	5
D'Avanzo,et al. 1995	1	0	0	1	1	1	0	1	0	5
Pujolar,et al. 1993 male	0	1	0	1	1	1	1	1	0	6
Pujolar,et al. 1993 female	0	1	0	1	1	1	1	1	0	6
Cole,et al. 1971 female	0	1	1	0	1	1	0	1	1	6
Cole,et al. 1971 male	0	1	1	1	1	1	0	1	1	7

* The study quality was assessed according to the Newcastle Ottawa Quality assessment scale for case-control studies. This scale awards a maximum of 9 points to each study: 4 for selection, 2 for comparability, and 3 for assessment of outcomes (for cohort study). 1 = "Yes", 0 = "No", "Unable to determine" or "Not available". For case-control studies, 1, indicates cases independently validated; 2, cases are representative of population; 3, community controls; 4, controls have no history of bladder cancer; 5A, study controls for age; 5B, study controls for additional factor(s); 6, ascertainment of exposure by blinded interview or record; 7, same method of ascertainment used for cases and controls; and 8, nonresponse rate the same for cases and controls.

Supplementary Table S4. Quality assessments of cohort studies on coffee consumption in relation to bladder cancer

Study	Selection				Comparability		Exposure			Overall quality score
	1	2	3	4	5A	5B	6	7	8	
	Indicates exposed cohort truly representative	Non-exposed cohort drawn from the same community	Ascertainment of exposure	Outcome of interest not present at start	Cohorts comparable on basis of age	Cohorts comparable on other factor(s)	Quality of outcome assessment	Follow-up long enough for outcomes to occur	Complete accounting for cohorts	
Kurahashi, et al.2009 male	1	0	1	1	0	1	1	1	1	7
Kurahashi, et al.2009 female	1	0	1	1	0	1	1	1	1	7
Michaud, et al. 1999	1	0	1	1	0	1	1	1	1	7
Tripathi, et al. 2002	0	0	1	1	0	1	1	1	0	5
Nagano, et al. 2000	0	0	1	0	0	1	1	1	0	4
Chyou, et al. 1993	1	0	0	1	0	1	0	1	1	5

* The study quality was assessed according to the Newcastle Ottawa Quality assessment scale for cohort studies. This scale awards a maximum of 9 points to each study: 4 for selection, 2 for comparability, and 3 for assessment of outcomes (for cohort study). 1 = “Yes”, 0 = “No”, “Unable to determine” or “Not available”. For cohort studies, 1, indicates exposed cohort truly representative; 2, non-exposed cohort drawn from the same community; 3, ascertainment of exposure; 4, outcome of interest not present at start; 5A, cohorts comparable on basis of age; 5B, cohorts comparable on other factor(s); 6, quality of outcome assessment; 7, follow-up long enough for outcomes to occur; and 8, complete accounting for cohorts.
